# Personality in Adults Who Were Born Very Preterm

**DOI:** 10.1371/journal.pone.0066881

**Published:** 2013-06-26

**Authors:** Christin L. Hertz, René Mathiasen, Bo M. Hansen, Erik L. Mortensen, Gorm Greisen

**Affiliations:** 1 Department of Neonatology, Copenhagen University Hospital, Rigshospitalet, Copenhagen, Denmark; 2 Institute of Public Health and Center for Healthy Aging, University of Copenhagen, Copenhagen, Denmark; Technical University of Dresden Medical School, Germany

## Abstract

**Aim:**

To investigate the personality in very preterm individuals (VPT; gestational age, GA, <32 weeks) at adult age in two cohorts born in 1974–76 and 1980–82, respectively, and to illuminate the effect of increased survival rates and the clinical implications of deviations in personality.

**Method:**

Demographic data were extracted for all individuals born in Denmark in 1974–76 and 1980–82. From each period one index-group each comprising 150 individuals with the lowest gestational age was selected. Thereafter two control groups born at term were matched by gender, age and residential area. Personality was assessed with the short version of NEO PI-R, and psychiatric diagnoses were obtained from the Danish Psychiatric Central Research Register.

**Results:**

Of all the individuals born <32 weeks of gestation in 1980–82 67% were alive in 2006 vs. 43% of those born in 1974–76 (p<0.0001). A total of 433 individuals participated in the study, 76% of the VPT groups (n = 227, mean GA = 27.9) and 69% of the control groups (n = 206). There were no significant differences on personality scores between the two VPT groups. Compared to the control groups, the combined VPT groups scored higher on neuroticism (p = 0.005) and agreeableness (p = 0.012), but lower on extraversion (p = 0.002). Psychiatric disorder was strongly associated with higher scores on neuroticism and lower scores on extraversion.

**Interpretation:**

Improved survival of VPT infants was not associated with increased deviances in the personality as adults. The personality traits in VPT individuals differ moderately from those of term born controls. High scores in neuroticism and low scores in extraversion were associated with increased risk psychiatric disorders. VPT adults also showed signs of positive adaptation in the form of an agreeable and confident attitude towards others.

**What this paper adds:**

The much improved survival rate in very preterm infants during the early years of active neonatology was not associated with increased risk of personality deviation. There are signs of positive adaptation in the form of increased agreeableness in young adults born very preterm.

## Introduction

Since the 1970s the number of surviving very preterm children (VPT children; <33 weeks of gestation) has increased significantly. In Denmark the proportion of children born VPT rose from approximately 0.7% in the mid-1970s to 1.2% in 2008 and during the same period mortality decreased from approximately 80% to 30% in the children born before 28 weeks of gestation and from approximately 40% to 4% in the children born from 28 to 31 weeks of gestation [1]. The growing number of VPT children has been followed with great interest. Follow-up studies from many different parts of the world have documented that VPT children have an increased risk of a wide range of developmental problems, such as neurosensory impairments, cognitive deficits, motor problems, behavioral problems and school difficulties [2], [3], [4], [5], [6]. Furthermore, elevated risk of psychiatric symptoms has been observed in teenagers who were born VPT [7] and recently an association between a low gestational age and psychiatric disorders in adults has also been found [8].

Thus studies investigating whether the wide range of reported difficulties in VPT children influence their adult lives are now being published, and a small number of these have investigated adult personality. Allin et al. found significantly lower extraversion and higher neuroticism and lie scores on the Eysenck Personality Questionnaire in VPT young adults born in the late 1980s compared to a group of term born controls [9]. The increased neuroticism and decreased extraversion scores were primarily observed in VPT women. Similarly Hack et al. found that very low birth weight (VLBW, birth weight <1500 grams) women born in the late 1970s, as adults, reported significantly more withdrawn behaviors than controls [10]. Pesonen et al. used the NEO PI-R self-report version and found that VLBW adults born in the late 1980s, irrespective of sex, scored higher on conscientiousness and agreeableness, but lower on openness to experience compared to a term born control group [11]. Schmidt et al. investigated a cohort from Canada of extremely low birth weight (ELBW; birth weight <1000 grams) born in the late 1970s and early 1980s [12]. They compared the ELBW adults without neurosensory and psychiatric disorders with a control group of normal birth weight. They found that young adults with ELBW were more cautious, shy and risk aversive and less extraverted. Thus, although the selection of the VPT cohorts and the methods investigating personality differ in the studies, there seems to be a picture of VPT individuals being less extraverted with increased neuroticism (i.e. more likely to experience anxiety, anger, guilt and clinical depression and often more self-conscious and shy). These characteristics in the personality in VPT individuals are related to psychopathological vulnerability but clinical implications of this such as increased incidence of psychiatric disorders have not been fully investigated. Furthermore, since there was an increase in survival of VPT children from the 1970s to the 1980s, more immature and probably sicker infants were surviving and in Denmark this was followed by an increase in the risk of cerebral palsy in the surviving infants born before 31 weeks of gestation [13]. This could also mean that an increased proportion of the survivors had other developmental difficulties including deviances in adult personality.

The aim of the present study was to further investigate personality in VPT adults and a potential influence of the increased survival. First, we examined whether VPT childreńs increased survival has been followed by deviations in personality at adult age by investigating two cohorts of VPT individuals born in Denmark in 1974–76 and 1980–82, respectively. Second, the influence of very preterm birth on personality was analyzed by comparing the personality of the VPT individuals with the personality of term born controls. Third, the possible clinical implications of any change in personality in VPT born adults were investigated by linking data on personality with data from a national register with information on psychiatric discharge diagnoses and by a questionnaire investigating health related quality of life.

## Methods

### Participants

All residents in Denmark are assigned a personal identification number (CPR-number) shortly after birth by the Danish civil registration system. This system was started in 1968. The CPR-number can be used to link various registries from Statistics Denmark, including registries with demographic and health information. Temporarily in a 5-year period from 2000 to 2006 the citizens were asked for permission to use their specific CPR number in non-anonymous research. Therefore some of the citizens, who were asked in this period, had declined that their CPR number can be used for non-anonymously research and researchers are not allowed to make a personal contact. However for anonymously research all individuals can be included.

**Figure 1:**
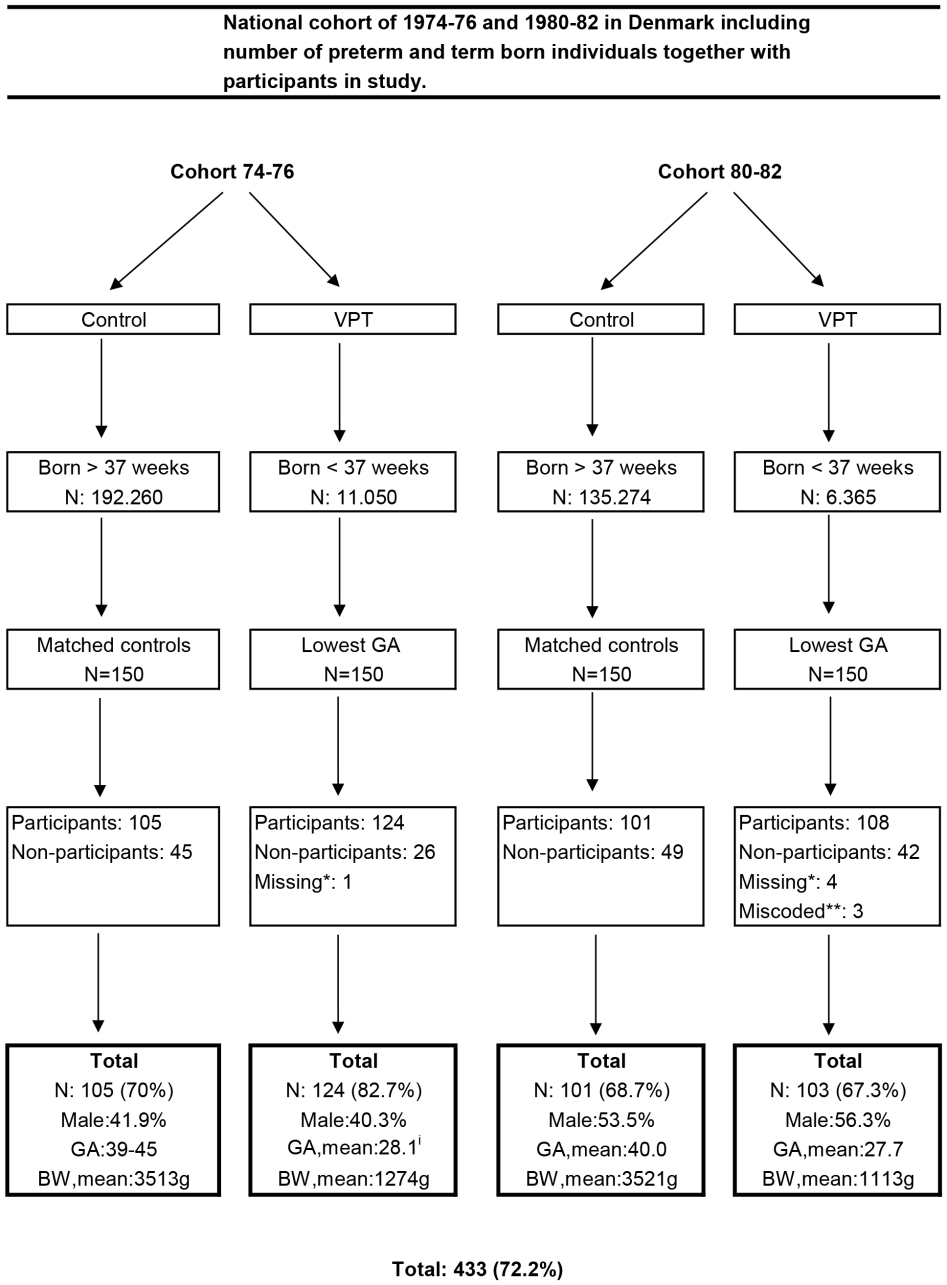
The flow diagram shows an overview of the selection, response rate and key data of the participants in the different cohorts. *birth weight data missing, **birth weight data not in accordance with estimated birth weight curve (Marsál), ^i^mean calculated from the grouping of gestational ages (e.g. group 29–31weeks approximated to 30 weeks). VPT, very preterm; GA, gestational age; BW, birth weight.

Two study cohorts born in 1974–76 and 1980–82 were selected. Among the individuals alive in 2006, 20.4% born in 1974–76 and 12.3% born in 1980–82 were registered as indisposed to participate in research. Thus these individuals could not be contacted personally, but anonymously data could be extracted from other databases. All individuals available for research born in the two periods were identified in the Fertility Register, and data on gestational age and birth weight were extracted. The individuals born in 1974–76 were registered in groups of gestational ages (e.g. 32–36 weeks, 29–31 weeks, ≤28 weeks) and the individuals born in 1980–82 were registered in full weeks of gestation. The values of birth weight and gestational age were evaluated by calculating a birth weight Z-score by the method described by Marsál [14], and three individuals with extreme values were excluded. The two cohorts were split in individuals born with a gestational age <37 weeks and a gestational age ≥37 weeks. From the two groups with a gestational age <37 weeks, the two index groups where selected. The individuals with the lowest gestational ages were identified, and 150 from each cohort were randomly selected. For each index group a control group of 150 individuals matched by gender, age and residential area was selected (Figure 1).

### Measures of Personality and Health Related Quality of Life

Personality was assessed with the Danish short version of the NEO PI-R and health-related quality of life (HRQOL) was measured using the Medical Outcomes Study Short Form 12 (SF-12). These two questionnaires were mailed to the members of the two cohorts. Like other versions of the NEO-PI-R, the Danish short version covers the five broad personality dimensions of neuroticism, extraversion, openness to experience, agreeableness and conscientiousness (Table 1) [15]. It is a self-report inventory comprising 60 items in a five-point Likert format with response categories ranging from “strongly disagree” to “strongly agree” [16]. The total raw scores are derived from 12 items scored 0–4 for each of the five factors and have a range from 0 to 48. Based on the Danish normative sample, the raw scores are converted to gender-specific normalized T-scores (range 22–78). For both the male and female normative samples the mean of the T-score is 50 and the standard deviation is 10 [17]. The SF 12 includes 12 items; two scale scores can be computed: the physical component summary (SF12-PCS) and the mental component summary (SF12-MCS). The mean is 50 in the general population, the range is 0–100, and higher scores indicate better QOL [18].

**Table 1:** The Personality Dimensions Measured by the NEO PI-R.

*Personality Dimension*	*Traits Measured*	*Main Attributes*
**Neuroticism**	anxiety, angry hostility, depression, self-consciousness, impulsiveness, vulnerability.	High neuroticism: more likely to experience anxiety, anger, guilt, clinical depression, and are often self-conscious and shy.
**Extraversion**	warmth, gregariousness, assertiveness,activity, excitement-seeking, positive emotions.	High extraversion: are social, impulsive, sensation seeking and have many friends. They may be more sexually active. Low extraversion: are quiet, introspective and keep their feeling to themselves.
**Openness**	fantasy, aesthetics, feelings, actions, ideas, values.	Low openness: are considered to be closed to experience, conventional and traditional in their outlook and behaviour. They prefer familiar routines and generally have a narrower range of interests. They could be considered practical and down to earth.
**Agreeableness**	trust, straightforwardness, altruism, compliance, modesty, tender-mindedness.	High agreeableness: have a optimistic view of human nature, tend to believe that most people are honest, decent, and trustworthy. Low agreeableness: are less concerned with others well-being, less likely to help other. They may be suspicious and unfriendly, have a tendency to be manipulative and are more likely to compete than cooperate.
**Conscientiousness**	competence, order, dutifulness, achievement striving, self-dicipline, deliberation.	High conscientiousness: are generally hard working and reliable. They may be workaholics, perfectionists, and compulsive in their behaviour. Low conscientiousness: tend to be more laid back, less goal oriented, and less driven by success.

The table elaborates the five different personality dimensions included in the personality test given to the participants.

The participants in the cohorts and the control groups were mailed the questionnaires in the period from June 2008 to October 2008, with thorough instructions for completing the questionnaires as well as an explanatory letter of the purpose of the study and their contributing part. The participants were recommended to complete the questionnaires alone, in a quiet and undisturbed environment without any time limit. The full guidelines for the tests were given to the participants. The age ranges of the participants in the two cohorts were 31–34 years and 25–28 year respectively, when they filled the questionnaires.

### Psychiatric Disorders

The index- and control groups were subsequently linked to the Danish Psychiatric Central Research Register and the Danish National Hospital Register which comprise information on all discharge diagnoses from secondary health care departments and make it possible to identify individuals with psychiatric disorders and individuals with cerebral palsy. These data are available for researches as there are no personal contacts and individuals are anonymous to the researcher. Since 1994 diagnoses have been coded according to the ICD-10 classification and before 1994 according to the ICD-8 classification. Cerebral palsy was identified using ICD-8 codes: 34.30–34.50 and ICD-10 codes: G80–G82.

To identify psychiatric disorders the following diagnostic categories were included: Any psychiatric disorders: (ICD-8∶29000–31600; ICD-10: F00.0–F99.9), Major psychiatric disorders including: ICD-8∶29509–29599, 29209–29299, 29419–29499, 29709–29899, 29909, 29309–29399, 29619, 29639, 29609, 29629; ICD-10: F20.0–F20.9, F23.0–F23.9, F30.0–F31.9, F32.0–F33.9. Minor psychiatric disorders including: ICD-8∶30100–30199, 29900, 29901, 30639, 30039, 30009–30029, 30049–30099, 29109–29199, 30309–30399, 30409–30499; ICD-10: F10.0–F10.9, F110–F199, F60.0–F60.9, F84.0, F98.5, F41.0–F419, F90.0–F90.9, F43.0–F43.9, F44.0–F48.9, and F42.0–F42.9. The psychiatric diagnoses were classified as major or minor, according to the hierarchical ICD coding including disorders with psychotic traits in major psychiatric disorders and other disorders in minor psychiatric disorders.

From the National Register of Education information on the level of education for both parents by 2007 was extracted, and the educational level of the parent with the highest level was classified into three categories according to the International Standard Classification of Education (ISCED) [19]. ISCED is based on the achieved qualifications rather than the length of the education: (1) those with basic education (compulsory basic education corresponding to 9 years in school, ISCED 0–2A); (2) skilled professionals (equivalent to secondary education, ISCED 3C-4C); and (3) those with tertiary education (different levels of professional education, ISCED 5A-6).

### Statistical Analysis

Tests of normality confirmed normal distributions for the scores on the NEO PI-R short version, and therefore the independent sample t-test was used to compare scores on the five personality dimensions between the index and control groups. The Mann-Whitney test was used to analyze the results from the SF 12.

The T-scores on the five personality dimensions (neuroticism, extraversion, openness to experience, agreeableness and conscientiousness) were used in multiple linear regressions. In univariate analyses we examined the explanatory variables: VPT vs. Term, gender, 1974–76 vs. 1980–82 cohort, psychiatric disease status and educational level of parents modelling the response variables of the five broad personality dimensions. Using the same variables a multiple linear regression model including all variables was performed.

The statistical package SPSS version 16.0 (SPSS Inc. Chicago, IL, US) was used.

### Ethics Approval

The study protocol was approved by the Danish Data Protection Agency and the National Board of Health. In Denmark studies based on questionnaires do not require approval of a research ethics committee.

## Results

### Survival, Participants and Personality of VPT Individuals in the Two Cohorts

The increased survival of VPT infants in Denmark from 1974–76 to 1980–82 was verified, thus 46.2% of the individuals born < = 28 weeks of gestation in 1980–82 were alive in 2006 vs. 21.5% of those born in 1974–76 (p<0.0001) and in the group of individuals born from 29 to 31 weeks of gestation there were 78.1% vs. 58.4% alive in 2006 (p<0.0001), respectively (Table 2). A total of 433 (72.2% of the entire study sample) individuals participated in the study, 75.7% of the individuals born preterm (n = 227) and 68.7% of the control groups (n = 206) (Figure 1). Mean gestational age for the VPT responders vs. non-responders was 27.9 (SD 1.0) weeks vs. 28.0 (SD 1.1) weeks (p = 0.5), and the corresponding mean birth weights were 1201 (SD 263.0) grams vs. 1219 (SD 245.9) grams (p = 0.130) (Table 3). Among the participants with incomplete self-reports on personality (n = 40, 9.4%) were 26 VPT individuals and 14 controls. Among the participants with incomplete SF 12 reports (n = 11, 3.0%) were 8 VPT individuals and 3 controls.

**Table 2:** Survivors of all infants born 1974–76 and 1980–82 in Denmark.

	GA	Total live births	Alive in 2006	
	weeks	N (100%)	N	(%)
**1974–76**	≤28	433	93	21.5
	29–31	639	373	58.4
	32–36	6686	6155	92.1
	≥37	199821	196662	98.4
**1980**–**82**	≤28	370	171	46.2
	29–31	668	522	78.1
	32–36	5937	5653	95.2
	≥37	136813	135255	98.9

The table shows survivors of all infants born in Denmark in the period from 1974–76 and 1980–82. The first column divides the infants in gestational age, the second column is the total of live births in the period, and the third column is the number of individuals alive in 2006. Data is from the National Board of Health, Denmark. GA, gestational age.

**Table 3:** Demographic data on birth, psychiatric disorders and cerebral palsy for participants and non-responders born in 1974–76 and 1980–8.

	Participants		Non-responders				All		
	VPT	control	VPT	control			VPT	control	
	N = 227	N = 206	N = 68	N = 94	p-value^a^	p-value^b^	N = 295	N = 300	p-value^c^
**BW (mean, gram)**	1201	3517	1219	3409			1210	3463	
**GA (mean, weeks)**	27,9	–^ i^	28,0	–^ i^			27,95	–^ i^	
**Psychiatric diagnosis all, N (%)**	27 (11.9)	10 (4.9)	10 (14.7)	9 (9.6)	ns	ns	37 (12.5)	19 (6.3)	<0.01
**Psychiatric diagnosis major, N (%)**	12 (5.3)	6 (2.9)	2 (2.9)	2 (2.1)	ns	ns	14 (4.7)	8 (2.7)	<0.01
**Psychiatric diagnosis minor, N (%)**	15 (6.6)	4 (1.9)	8 (11.8)	8 (8.5)	<0.05	ns	23 (7.8)	12 (4)	<0.01
**Cerebral palsy, N (%)**	13 (5.7)	0 (0.0)	11 (16.2)	0 (0.0)	<0.05	–	24 (8.1)	0 (0.0)	<0.01

The table shows demographic data for the participants and non-responders subdivided into VPT and termborn controls. The last comparison comprises all individuals in both cohorts.

** p<0.01;

* p<0.05; ns = not significant;

a comparison of participants and non-responders born VPT;

b comparison of participants and non-responders in the control group;

c comparison of all VPT and controls; i GA = 39–45; BW, birth weight; GA, gestational age; VPT; very preterm.

There were no significant differences on personality scores between the two VPT cohorts, while in the control groups conscientiousness were higher in the 1974–76 than in the 1980–82 cohort, 54.9 vs. 51.3 (p = 0.02).

### Personality of VPT vs. Term Born Participants

Since there were no differences between the two VPT cohorts in terms of the personality test, the two index groups were analyzed together. The combined VPT and control groups differed significantly with respect to three of the five personality dimensions (Table 4). The VPT group scored higher on neuroticism (p = 0.005) and agreeableness (p = 0.012), but lower on extraversion (p = 0.002). The size of the differences in means between the VPT and controls corresponded to 0.2–0.4 standard deviation.

**Table 4:** Mean T-score of the different dimensions in NEO PI-R and SF12 within the 1974–76 and the 1980–82 cohorts.

	Cohort 1974–1976					Cohort 1980–82					VPT					All				
	VPT		Control			VPT		Control			1974–76		1980–82			VPT		Control		
	(N = 124)		(N = 104)			(N = 103)		(N = 101)			(N = 124)		(N = 103)			(N = 227)		(N = 205)		
	Mean	(SD)	Mean	(SD)	p-value	Mean	(SD)	Mean	(SD)	p-value	Mean	(SD)	Mean	(SD)	p-value	Mean	(SD)	Mean	(SD)	p-value
**Neuroticism**	54,2	(12.4)	50.1	(12.2)	*	55.1	(12.4)	52.7	(10.4)	ns	54	(12.4)	55.1	(12.4)	ns	54.6	(12.4)	51.4	(11.4)	**
**Extraversion**	48.0	(12.0)	52.7	(11.7)	**	50.2	(13.7)	52.2	(9.4)	ns	48.0	(12.0)	50.2	(13.7)	ns	49.0	(12.8)	52.5	(10.6)	**
**Openness**	51.3	(10.6)	53.5	(10.6)	ns	53.2	(10.1)	53.6	(9.3)	ns	51.3	(10.6)	53.2	(10.1)	ns	52.1	(10.4)	53.5	(9.9)	ns
**Agreeableness**	51.8	(9.6)	50.8	(10.8)	ns	51.9	(10.3)	47.9	(10.8)	**	51.8	(9.6)	51.9	(10.3)	ns	51.9	(9.9)	49.3	(10.9)	**
**Conscientiousness**	52.7	(11.9)	54.9	(10.9)	ns	52.4	(13.4)	51.3	(11.1)	ns	52.7	(11.9)	52.4	(13.4)	ns	52.5	(12.6)	53.1	(11.1)	ns
**SF12-PCS**	52.3	(7.6)^a^	52.3	(8.1)^a^	ns	52.4	(7.5)^b^	53.6	(6.2)^b^	ns	52.3	(7.6)^a^	52.4	(7.5)^b^	ns	53.4	(7.6)^c^	52.9	(7.2)^c^	ns
**SF12-MCS**	50.6	(9.7)^a^	52.0	(8.3)^a^	ns	49.7	(10.6)^b^	51.4	(8.7)^b^	ns	50.6	(9.7)^a^	49.7	(10.6)^b^	ns	50.2	(10.1)^c^	51.7	(8.5)^c^	ns

The table shows the mean scores for the personality test and the test for health related quality of life, comparing each cohort separately, the VPT individuals and finally all participants.

** p<0.01;

* p<0.05; ns = not significant;

a VPT (N = 119), control (N = 104);

b VPT (N = 99), control (N = 99);

c VPT (N = 218), control (N = 203);

d VPT (N = 194), control (N = 191); VPT, very preterm; SF12-PCS, Short Form 12- Physical Component Summary; SF12-MCS, Short Form 12-Mental Component Summary.

The personality dimensions were investigated further by regression analyses (Table 5). There were significant associations between personality traits and parental education in univariate analyses but in the multivariate models parental education was only significantly associated with scores on extraversion (p = 0.004). There was, as expected, no gender effect in the univariate analyses because of the T-transformation of the scores. In the multivariate analyses there were significantly higher conscientiousness scores in women (p = 0.036), but since there was no interactions with respect to any of the five broad personality dimensions this was not a specific finding for the VPT cohorts. The differences on neuroticism, agreeableness and extraversion between the VTP and the controls could not be explained by the variables parental education, psychiatric diseases, period of birth and gender as shown in the multiple regression analyses. The multivariate model showed no significant differences between the 1974-76 cohorts and the 1980–82 cohorts (Table 5).

**Table 5:** Multiple regression analyzis of the personality dimensions measured by NEO-PIR of survived VPT and control group individuals born in 1974–76 and 1980–82.

	Neuroticism				Agreeableness				Conscientiousness				Extraversion				Openness			
	Univariate		Multivariate		Univariate		Multivariate		Univariate		Multivariate		Univariate		Multivariate		Univariate		Multivariate	
Parameter	Estimate		Estimate		Estimate		Estimate		Estimate		Estimate		Estimate		Estimate		Estimate		Estimate	
Intercept			**61,46**	***			**50,55**	***			**44,87**	***			**48,05**	***		***	**58,16**	***
Very preterm vs. Term	**3,26**	**	**2,53**	*	**2,53**	*	**2,40**	*	**−0,60**	**ns**	**0,16**	**ns**	**−3,50**	**	**−2,63**	*	**−1,39**	**ns**	**−1,40**	**ns**
Parent ISCED low vs. high	4,24	*	**3,72**	ns	−0,45	ns	−0,71	ns	**−1,68**	ns	−1,36	ns	**−9,49**	***	−8,89	**	−5,60	**	**−5,57**	ns
Parent ISCED middle vs. high	3,22	*	**3,22**	ns	−2,66	*	−2,77	ns	**−2,32**	ns	−2,59	ns	**−3,48**	**	−3,49	ns	−1,92	ns	**−2,18**	ns
Psychiatric disease no vs. yes	64,68	***	**−11,37**	***	−0,38	ns	−0,08	ns	**10,42**	***	10,23	***	**8,40**	***	7,77	***	−1,60	ns	**−1,73**	ns
1974–76 vs 1980–82 cohort	−1,57	ns	**−2,18**	ns	1,42	ns	1,22	ns	**1,83**	ns	1,79	ns	**−1,07**	ns	−0,52	ns	−1,15	ns	**−1,12**	ns
Male vs. Female	−0,79	ns	**−0,22**	ns	−0,80	ns	−1,03	ns	**−1,96**	ns	−2,40	*	**0,52**	ns	−0,23	ns	−1,50	ns	**−1,72**	ns

The table shows the multiple regression analyzes of the personality test including parental education, psychiatric disease, age and gender.

*** = <0.0001,

** = <0.005,

* = <0.05, ns = not significant. Multivariate models adjusted for: educational level of cases, educational level or parents, psychiatric disease status of parents, sex and cohort year. *ISCED*, International Standard Classification of Education; VPT, very preterm; SF12-PCS, Short Form 12- Physical Component Summary; SF12-MCS, Short Form 12-Mental Component Summary.

### HRQOL and Psychiatric Disorders

Concerning HRQOL all results had a mean around 50 (Table 4). There was no difference in HRQOL mean score between participants born preterm vs. controls, neither for the physical component, mean 53.4 vs. 52.9 (p = 0.6), nor the mental component, 50.2 vs. 51.7 (p = 0.2). Comparing the 1974–6 and the 1980–2 cohorts, there were no differences in the scores in the two components.

Among the 433 participants answering the self-reported questionnaires, nine percent had one or more psychiatric diagnoses. Psychiatric diseases were strongly associated with higher scores on neuroticism and lower scores on conscientiousness and extraversion shown in the multivariate analyses (Table 5). The cumulated incidence of any psychiatric disorders was higher in VPT individuals (12.5% vs. 6.3%, p = 0.0084) and so was the incidence of cerebral palsy (8.1% vs. 0.0%, p = 0.0001). There was no difference in the incidence of any psychiatric disorders (11.9% vs. 14.7%, p = 0.457) between the participating and non-participating VPT groups, while the incidence of cerebral palsy was higher in the non-participating (16.2%) compared to the participating (5.7%) VPT group (p = 0.004) (Table 3). Concerning the incidence of psychiatric disorders and cerebral palsy we found no differences between the non-participants VPT individuals from 1974–1976 and 1980–82 (data not shown).

## Discussion

Our study aimed to extend the previous research investigating how personality in adulthood is influenced by VPT birth.

First we investigated whether the increase of the survival rate of VPT infants was followed by increased deviances in personality as adults. We found no differences in the scores in the personality test between the two VPT cohorts. Personality is likely to arise from interaction between multiple genetic and environmental factors [20]. Previous studies found that VPT individuals have deviances in personality compared to term born individuals and one etiological factor for this could be early brain injury and in this perspective we would have expected to find a difference between the two VPT cohorts. We did not investigate association between perinatal data and personality accordingly we can only speculate that one explanation for our finding maybe that the improvements in perinatal treatment and care in the cohort from the 1980s resulted in both increased survival and better long-term outcome in the survivors.

Second we found that compared to term born controls VPT individuals had significantly higher scores on neuroticism, lower on extraversion and higher on agreeableness than the term born adults. Overall this finding are in line with those reported previously. Thus, in UK Allin et al [9] found significantly lower extraversion and higher neuroticism and lie scores on the Eysenck Personality Questionnaire in VPT young adults compared to a group of term born controls, and in the US Hack et al. [10] found that VLBW women reported significantly more withdrawn behaviors than control subjects as adults. These personality deviations are similar to our findings, but in contrast to our results these two studies observed gender differences. However, the transformation to gender-specific normalized T-scores in our study seems to have eliminated most gender specific differences. In Finland, Pesonen et al. [11] used the NEO PI-R and compared VLBW young adults with term-born controls. They found higher scores on conscientiousness, agreeableness and lower openness to experience and additionally the VLBW group scored different from the controls with regard to facets of neuroticism (lower hostility and impulsivity) and extraversion (less assertiveness). We did not find a significant difference with respect to conscientiousness or openness to experience, but the differences between VLBW and controls on neuroticism, extraversion and agreeableness were similar to our findings. Finally Schmidt et al. [12] compared a cohort from Canada of ELBW adults without neurosensory and psychiatric disorders with a control group of normal birth weight adults. They found that young adults with ELBW were more cautious, shy and risk aversive and less extraverted which is in agreement with the lower scores on extraversion in our study. In our study the differences in the scores of the personality test between the VPT and the term born control group corresponded to 0.2–0.4 standard deviation and compared to the IQ deficit found in the VPT cohorts of at least 0.7 standard deviation this difference is moderate [21], [22]. Our data suggest that the development of personality is less affected by very preterm birth than the development of intelligence and our finding implies that the deviances of personality must be negligible in many of the VPT adults.

Third we investigated the clinical significance of the typical deviances in the personality in VPT individuals. We found that higher neuroticism and lower extraversion scores, observed in VPT adults, were associated with psychiatric diseases. This finding is in accordance with other recent studies also finding increased incidence of psychiatric disorders in VPT adults [8], [23]. Although our data support that VPT adults have increased psychopathological vulnerability we found that measures of health-related quality of life were similar in the index and control groups. This finding is more controversial and we believe that the major reason for the different findings is related to the method of how to measure the health-related quality of life [24].

The validity of a cohort study is always sensitive to how representative the cohort is for the whole population and loss at follow-up. We believe that our study has major strengths: The national character of the two VPT cohorts combined with an acceptable response rate provides population based data. Furthermore, we were able to investigate whether the results were biased due to selective loss of individuals with psychiatric disorders which appeared not to be the case. We consider it likely that the individuals with cerebral palsy who did not participate in the study had cognitive impairments so that they were not able to answer the questionnaires. However since the number of individuals with cerebral palsy was low this selection bias could not affect the overall results. To reduce the influence of sociodemographic factors, the control groups were matched to the index groups on residential area, besides age and gender. Parental education was also controlled, but it was not possible to include the parental mental health. The NEO PI-R and the SF12 questionnaires are well-established questionnaires, and Danish normative data are available. The Danish short version of the NEO PI-R has been demonstrated to be highly correlated with the original full-length questionnaire, and consequently we used this version to facilitate a higher response rate.

Overall our study corroborates the picture of VPT individuals being less extraverted with increased neuroticism as adults and this finding seems remarkably consistent in the literature. The increase in agreeableness in the VPT group, which also was found by Pesonen et al. [11], may be a sign of positive adjustment. This finding could help explaining the results of a recent Danish register-based study showing that the vast majority of VPT individuals born in 1974–76 actually seem well integrated in society at age 27–29 years [25]. The increase in agreeableness appeared to be unrelated to the educational level of the parents, but maybe environmental factors such as increased parental sensitivity observed in a controlled study of VPT children at pre-school age [26] could explain our finding of higher scores on agreeableness. We speculate that early parental adjustment to their very preterm child may have long-term benefits in helping the child to develop an optimistic attitude and confidence in others that are positively associated with adjustment in adult life. We were not able to investigate associations between early morbidity, parental factors and personality in this study, but future studies should try to illuminate these associations.

Even though the significantly increased survival in the 1980–82 cohort in our study was not related to significant differences in personality in the VPT adults compared to the 1974–76 cohort, the number of individuals born before 26 weeks of gestation was very low in our cohorts so our results should not be generalized to the most immature infants of only 23 to 25 weeks of gestation surviving today.

### Conclusions

The personality traits in VPT individuals differ moderately from those of term born controls; VPT individuals are less extraverted and show increased neuroticism as adults. This finding seems remarkably consistent and at the group level the observed VPT personality characteristics are related to vulnerability to psychopathology. Importantly, and perhaps less anticipated, VPT adults also show positive signs of adaptation in the form of a positive and confident attitude towards others.
